# Bacteriophages from ExPEC Reservoirs Kill Pandemic Multidrug-Resistant Strains of Clonal Group ST131 in Animal Models of Bacteremia

**DOI:** 10.1038/srep46151

**Published:** 2017-04-12

**Authors:** Sabrina I. Green, Jason T. Kaelber, Li Ma, Barbara W. Trautner, Robert F. Ramig, Anthony W. Maresso

**Affiliations:** 1 Molecular Virology and Microbiology Department, Baylor College of Medicine, Houston, TX 77030, USA; 2 Michael E. Debakey Veterans Affairs Medical Center, Houston, TX, 77030, USA

## Abstract

Multi-drug resistant (MDR) enteric bacteria are of increasing global concern. A clonal group, *Escherichia coli* sequence type (ST) 131, harbors both MDR and a deadly complement of virulence factors. Patients with an immunocompromised system are at high risk of infections with these *E. coli* and there is strong epidemiologic evidence that the human intestinal tract, as well as household pets, may be a reservoir. Here, we examine if phages are an effective treatment strategy against this clonal group in murine models of bacteremia that recapitulate clinical infections. Bacteriophages isolated from known *E. coli* reservoirs lyse a diverse array of MDR ST131 clinical isolates. Phage HP3 reduced *E. coli* levels and improved health scores for mice infected with two distinct ST131 strains. Efficacy was correlated to *in vitro* lysis ability by the infecting phage and the level of virulence of the *E. coli* strain. Importantly, it is also demonstrated that *E. coli* bacteremia initiated from translocation across the intestinal tract in an immunocompromised host is substantially reduced after phage treatment. This study demonstrates that phage, isolated from the environment and with little experimental manipulation, can be effective in combating even the most serious of infections by *E. coli* “superbugs”.

Extraintestinal pathogenic *E. coli* or ExPEC are a broad *E. coli* pathotype that asymptomatically colonize the human and canine gastrointestinal tract but are capable of infecting sites distal to the intestine^1^,^2^. An ExPEC clonal group, referred to as sequence type 131 (ST131), has emerged as a pandemic multidrug-resistant (MDR) pathogen capable of producing extended spectrum beta lactamases, is refractory to fluoroquinolones, and is widely distributed in the community and hospital settings^3^,^4^,^5^. In addition to carrying resistance elements, these strains also harbor a deadly compliment of virulence factors^6^,^7^,^8^. This single clonal group is now considered the predominant cause of all antibiotic resistant *E. coli* infections in the US, with this species now the most frequently isolated Gram-negative bacterium from the blood of adult patients with sepsis^9^,^10^. Sepsis was associated with 6% of all deaths in the US during 1999–2014 and one fourth of these deaths attributed to infections originating from the urinary or gastrointestinal tract^9^,^11^. With 750,000 sepsis cases occurring annually in the US alone, and at a cost of more than $20 billion per year, there is an unmet need for new and effective treatments to combat drug-resistant bacterial infections^12^,^13^.

One possible solution to this growing issue is to develop bacteriophages (phages), natural parasites of bacteria, as prophylactic or therapeutic agents to kill resistant strains^14^. Bacteriophages were first described by F. W. Twort in 1915^15^. Félix d’Herelle later characterized them as lytic agents, capable of self-amplification on a suitable bacterial host, and developed the idea for phage therapy as a way to control infections caused by bacteria^16^,^17^. With the advent of antibiotics in the 1940s, the prospect of phage therapy was discarded in the West, but several Eastern European nations have successfully used this approach to treat a variety of bacterial infections^17^,^18^,^19^,^20^. The rise of antibiotic resistance in several bacterial pathogens that cause serious infections in humans has reinvigorated phage research with new forays into safety and clinical phase trials involving venous leg ulcers^21^, burn wounds^22^, ear infections^23^, and diarrhea^24^. Clinical trials show some success with drug-resistant bacterial infections^23^,^25^ with little to no adverse effects upon phage administration^21^,^25^. The rigid process by which new drugs are approved, the high level of costs associated with bringing new medicines to the market, and the lengthy time frame these processes typically require implies the development of new antibiotics may not keep pace with the constantly evolving and adapting nature of the bacterial world. Phages by their very nature are adaptive and thus can be tailored to the clinical needs at hand. Working with this premise in mind, we report here the isolation, characterization, and evaluation of the efficacy of phage in relevant animal models of ExPEC infection, thereby laying the groundwork for the study of phage as an alternative strategy to contend with the rise of MDR organisms.

## Results

### Isolation of phage that kill ExPEC sequence type 131

**Figure 1:**
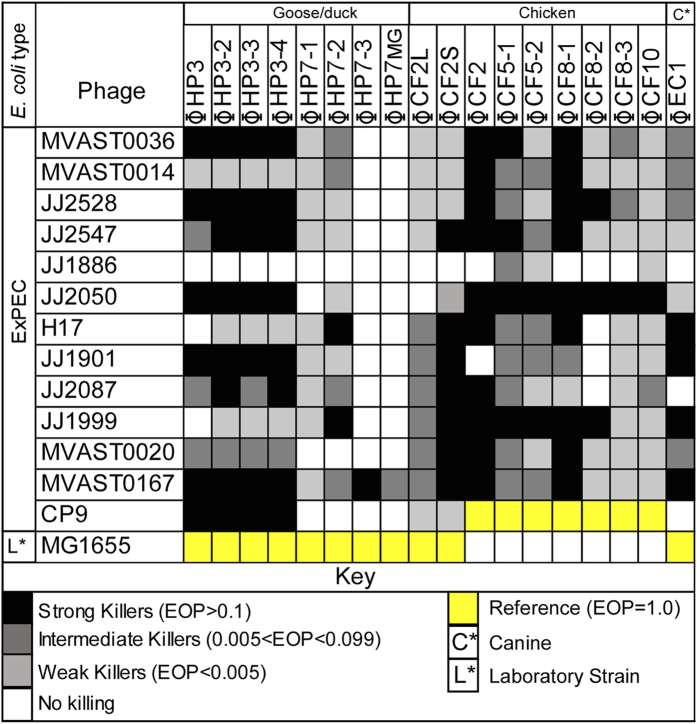
Lytic activity of isolated phages on ST131 ExPEC. The killing potential of each phage was determined by calculating the efficiency of plating, or EOP, on each *E. coli* strain to strain MG1655 (colored yellow) or CP9 (colored yellow) which was set to 1. Dark black squares indicate, EOP > 0.1, dark grey 0.005 < EOP < 0.099, light grey EOP < 0.005 and white, no lysis observed.

Phages are ubiquitous in the environment. Due to the epidemiologic link in the virulence factor composition between avian pathogenic *E. coli* (APEC)^2^, canine *E. coli* that are positive for *pap* (pyelonephritis-associated pili)^26^, and the known virulence factor profile of human ExPEC, some of which have been documented in these reservoirs^26^, we sought to identify lytic phage from these *E. coli* reservoirs (see Methods). Using either a well-characterized ExPEC strain (CP9) or the laboratory strain *E. coli* MG1665 as the host, each reservoir yielded multiple plaques upon plating. Plaques from both hosts were tested for lytic activity on a panel of drug resistant ST131 ExPEC isolates^27^ with broad virulence properties and clinical origin. The three phage reservoirs produced phages collectively capable of lysing every strain. Distinct specificities were noted for each phage isolate. All reservoirs produced virus capable of infecting >90% of the ExPEC strains except derivatives of ϕHP7 which showed overall lower activity compared to ϕHP3 (<10%) (Fig. 1). Interestingly, JJ1886 was resistant to all phage except CF5-1, CF5-2, and CF10, which only weakly killed this ExPEC. Only MVAST0167 was killed by every phage.

### Phage characterization and morphological analysis

We sought to characterize some of these phages for the presence of toxins or lysogenic elements, their basic infection parameters, and examine their morphology for classification. We chose to first sequence phages HP3, EC1, and CF2 because they were each isolated from a different source, they each display quite distinct strain specificity for their host bacterium (Fig. 1), and they are likely to be phages pursued for future studies. Phages were sequenced by applying a DNA library generated by ligation mediated-PCR (LM-PCR) to an illumina MiSeq platform (see Methods). Phage HP3 is 168,188 base pairs in size with 274 predicted open reading frames. Phage CF2 is 53,242 base pairs in size with 74 predicted open reading frames while that of EC1 is 170,254 base pairs with 275 ORFs (Table 1). Interestingly, phage HP3 is highly similar to Shigella phage pSs-1^28^ with approximately 96% homology between the two for ORFs present in both phages. Phage CF2 is highly similar to Salmonella phage BP63 (accession # NC_031250.1, 96% over shared ORFs). Phage EC1 is similar to Shigella phage SHSML-52-1 (98% identity, accession # KX130865.1). BLASTP searches were performed on predicted ORFs for a functional analysis and all virulence genes were searched using VirulenceFinder^29^. No virulence genes of known toxicity (viral or bacterial) or genes involved in lysogeny (*e.g*. lysogeny cassettes) were detected. Mitomycin C treatment of *E. coli* strain JJ2528 infected with phage HP3, using the method described for Zeng *et al*.^30^ which in our hands induced lysogeny of lambda phage (10,000 PFU/mL) from *E. coli* MG1655, did not yield any phage, confirming that HP3 is a lytic phage (data not shown). In addition, no genes that encode resistance to known antibiotics, as assessed using ResFinder^31^, were identified in HP3, CF2, or EC1. Thus, it seems that these phages are void of any known lysogenic or toxic elements that would preclude their use in experimental studies of efficacy.

**Figure 2:**
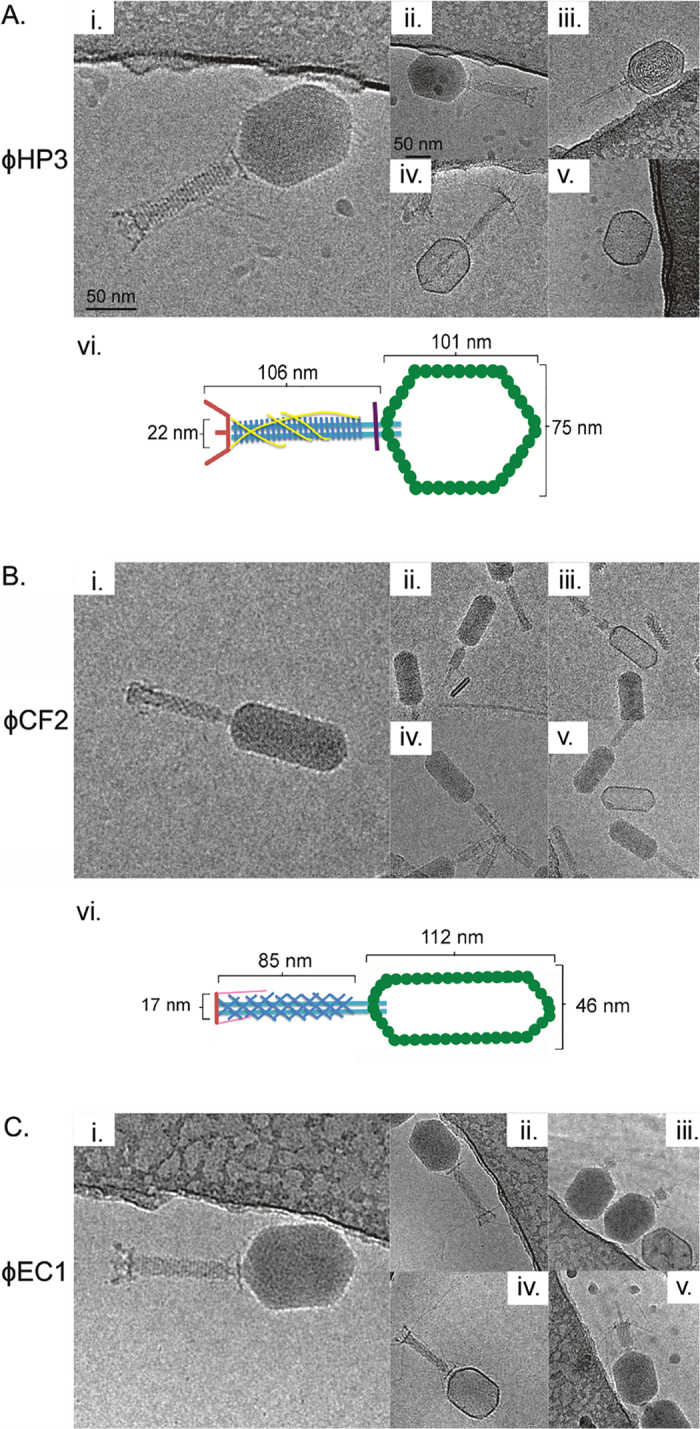
Cryo-EM imaging of bacteriophages. (**A**) Purified HP3 virus (i) including dimensions (vi) with extended tail fibers (ii and iv), tail contracted (iii) and with DNA absent (v). (**B**) CF2 virus (i) including dimensions (vi) with tail contracted (ii), DNA absent (iii), DNA injecting (iv) and DNA and tail absent (v). (**B**) EC1 virus (i) with tail fibers extended (ii and iv), DNA injecting (iii), and tails contracted (v).

Along these lines, we measured the (i) adsorption rate, (ii) burst size, and (iii) plaque morphology and size for HP3 (Table 2). The mean adsorption rate constant was determined to be 5.63 × 10^−9^ mL/min with 99.8% adsorbed during a 20 minute infection period. The mean burst size and latent period were determined to be 60 and 22.5 min, respectively. All plaques had a mean diameter of 0.5 mm and were clear. The morphological properties of the phages were determined by cryoelectron microscopy. ϕHP3 (Fig. 2A) contains a prolate icosahedral head (W 75 nm, L 101 nm) and a clearly discernible T-4 like tail (W 22 nm, L 106 nm). The base plate was visible and the tail fibers, when visible, were observed in both the extended and contracted position (Fig. 2A,i and iv). A small proportion of particles lacked DNA (Fig. 2A,v). ϕCF2 contains a tail of similar dimensions but with oblique striations (W 17 nm, L 85 nm). Its thin head (W 46 nm) gives it an overall elongated appearance (Fig. 2B). ϕCF2’s capsid was substantially prolate and the tail fibers were only visible in particles with the tail in the contracted position (Fig. 2B,ii and iv). A small number of empty particles were observed with the DNA ejected (Fig. 2B,iii) or not properly packaged (Fig. 2B,v). The particularly prolate capsid of ϕCF2 is similar to some viruses of the family *Siphoviridae*^32^. Phage EC1 was similar to ϕHP3 in morphology (Fig. 2C). All three phage displayed features consistent with the family *Myoviridae*. The ability of the tail to contract, diagnostic of *Myoviridae*^33^, is supported by the presence of a minority of virions with partially or fully contracted tail sheaths (Fig. 2A,iii,B,ii and C,v). The tail inner tube extends past the end of the tail sheath in those cases. Curvature in the tail is often seen in *Siphoviridae*, but was never observed in the phages imaged here.

### Therapeutic efficacy of phage with activity towards ST131

**Figure 3:**
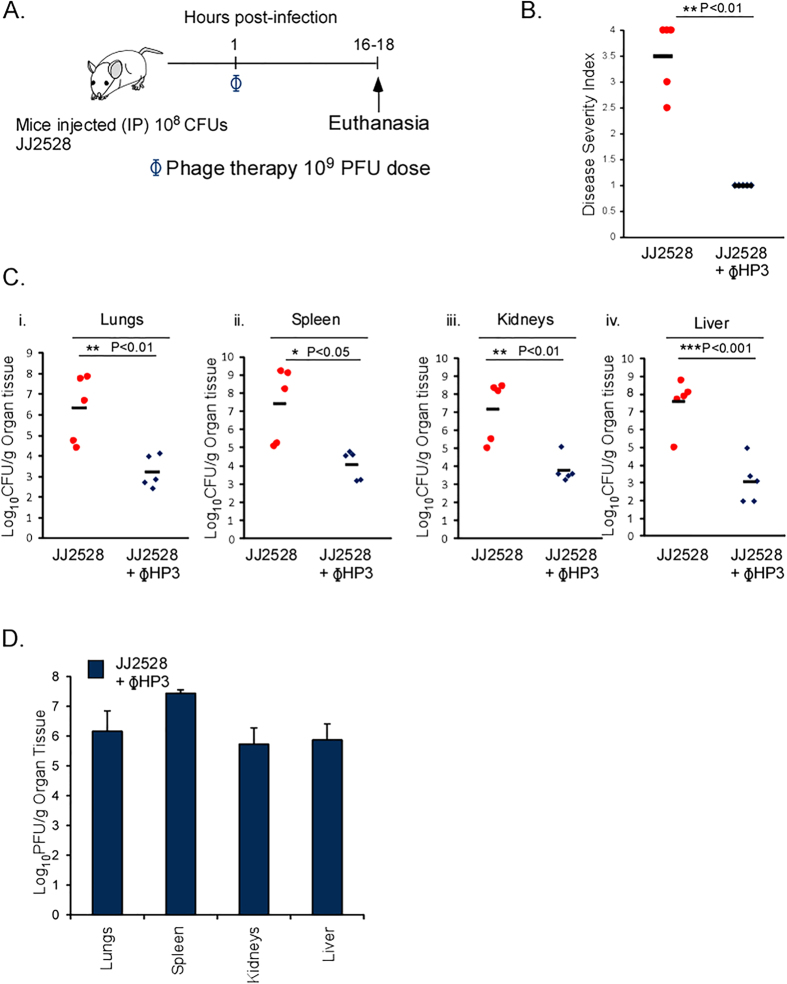
Phage therapy in a mouse model of ST131 MDR bacteremia. (**A**) BALB/c, 6 week old, female mice were infected with an intraperitoneal (IP) injection of 10^8^ CFU of JJ2528. One hour following infection mice were injected IP with 10^9^ PFUs of purified ΦHP3. Disease severity was assessed and organs harvested and plated to determine bacteria and phage levels. (**B**) Disease severity of mice following an 18 hour infection. Red dots represent individual mice given bacteria alone and blue diamonds represent the phage treated, infected mice. Black bars represent the mean values for each group. (**C**) Organs CFU of JJ2528. (**D**) Organ CFU of ΦHP3 plaqued on JJ2528. Panel B, p-values generated using Wilcoxon rank sum test. Panels C,D, p-values were generated using a T-test. One star (*) p < 0.05, two stars (**) p < 0.01, and three stars (***) p < 0.001. Error bars represent the standard deviation and n is equal to 5.

ExPEC ST131 strains are drug resistant and comprise a significant worldwide burden of bloodstream infections. We therefore studied whether the phages isolated and characterized here display therapeutic efficacy in a systemic model of ExPEC bacteremia. Mice were infected with a virulent ST131 strain, JJ2528 (10^8^ CFU), which is sensitive to ϕHP3, via the intraperitoneal route. One hour later, the infected animals were treated with ϕHP3 (10^9^ PFU) and disease monitored after 18 hours (Fig. 3A). Infected mice given ϕHP3 were substantially healthier (mean score of 1) than infected groups that did not receive phage (mean score 3.5) (Fig. 3B). Animals receiving ϕHP3 demonstrated, on average, a 3–4 log reduction in the bacterial burden across all organs examined (Fig. 3C). Phage were likewise recovered from these tissues (Fig. 3D).

**Figure 4:**
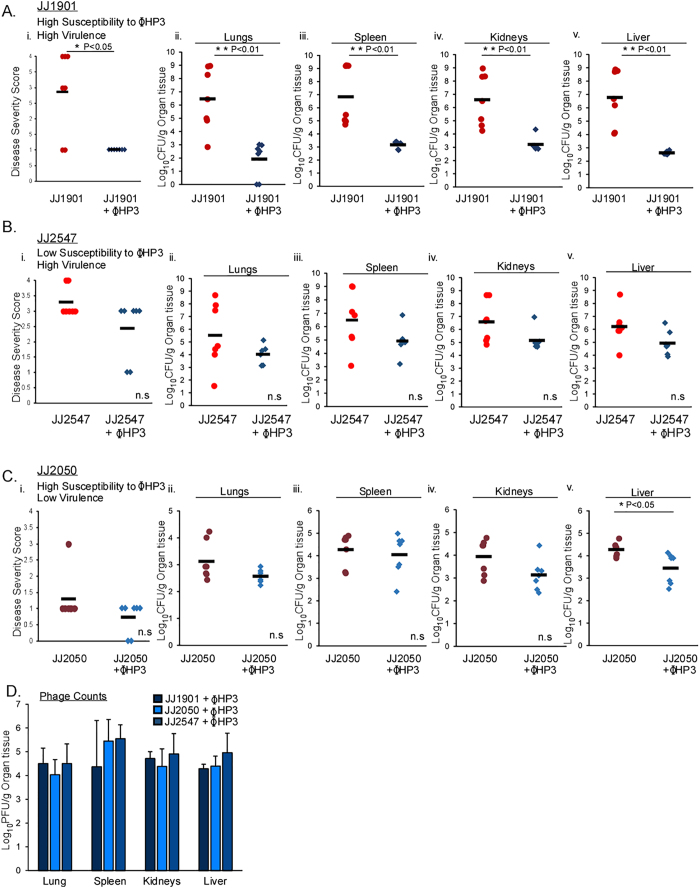
Comparison of phage therapy dynamics in a mouse model of ST131 bacteremia. (**A**–**D**) Swiss Webster, 6 week old, female mice were infected with various ST131 strains and treated as described in Fig. 3. Susceptibility was determined by the EOP values in Fig. 1. (A–C) Dark red, maroon and red dots represent individual mice injected with bacteria alone and dark blue, blue and light blue diamonds infected mice treated with phage ΦHP3. Black horizontal lines indicate the mean values of groups. (i) Disease severity scores and (ii–v) organ counts were assessed as described in the Methods. (**D**) Blue bars represent mean values of PFU/g in phage- treated mice. The letters n.s. indicate no statistical difference. (**A**–**C**) (i) p-values generated using Wilcoxon rank sum test, (**A**–**C**) (ii–v) p-values were generated using a T-test. (**D**) p-values using One-way ANOVA and Tukey’s test (no significance, not shown). *p < 0.05, **p < 0.01, and ***p < 0.001. Error bars represent standard deviation and n is equal to 7.

Next, we sought to explore the effectiveness of ϕHP3 relative to two key parameters important for therapeutic phages; (i) specificity of the phage amongst various *E. coli* ST131 strains in the context of an infection and (ii) the virulence of the bacterial strain in relation to the efficacy of the phage in the mammalian, infected host. We first tested the *in vivo* specificity of ϕHP3 by examining its efficacy against two strains, JJ1901 and JJ2547, both of which demonstrate high virulence but variable susceptibility to this phage (Fig. 1). Mice were infected as described in Fig. 3A and the health or bacterial levels in the liver, spleen, kidney, and lungs were measured. Mice infected with JJ1901 showed a similar course of disease and dissemination to organs as JJ2528, consistent with it being a virulent strain (Fig. 4A,i–v). Similar to JJ2528, when infected with JJ1901 and then given a single dose of ϕHP3, both the overall health scores as well as the levels of ExPEC in the tissues were markedly reduced (~3.5 logs) (Fig. 4A,i–v). However, when animals were infected with strain JJ2547, another highly virulent ExPEC strain with similar health and dissemination parameters as JJ1901 but low sensitivity to ϕHP3, phage treatment demonstrated a modest improvement in health scores and lowering of ExPEC levels (only ~1 log reduction), both of which were not statistically significant (Fig. 4B,i–v). We next tested the therapeutic efficacy of ϕHP3 towards a strain that is highly susceptible to this phage in culture (JJ2050, Fig. 1) but displays low intrinsic virulence in the murine model. As expected, strain JJ2050 did not induce significant disease (score of 1.2) or attain high levels of bacterial burden in the surveyed organs (~3 logs less than JJ1901) in this model (Fig. 4C,i–v). Interestingly, despite being highly susceptible to ϕHP3 in culture, infected animals treated with ϕHP3 showed a non-significant reduction of only 1.25 logs of CFU in major organs, which is in contrast to the ~3–4 logs observed for strains JJ2528 and JJ1901. All three infections yielded abundant and similar levels of ϕHP3 in the lungs, spleen, kidneys, and liver.

### The efficacy of phage in neutropenic hosts undergoing bacterial translocation

**Figure 5:**
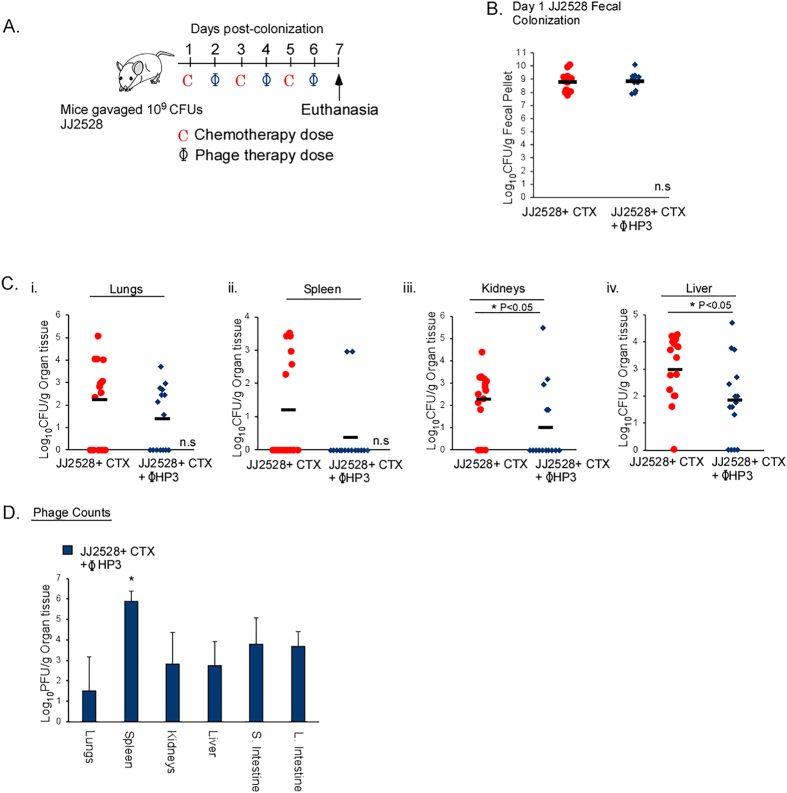
Efficacy of phage therapy in an immunocompromised-mouse model of bacterial translocation. (**A**) Swiss Webster, 6 week old mice were gavaged with 10^9^ CFU of JJ2528 on day 0, then treated with the chemotherapeutic agent cytoxan (CTX), IP. The phage HP3 (10^9^ PFU per dose) was given IP separately on days 2, 4 and 6 post-gavage. On day 7, organs were harvested for plating of bacteria and plaquing of phage. (**B**) Fecal pellets were collected, homogenized and plated on LB plates with ampicillin. Red circles represent individual mice gavaged with JJ2528 and treated with cytoxan. Blue diamonds represent individual mice gavaged with JJ2528 and treated with CTX plus HP3 phage. Horizontal black lines represent the mean. (**C**) Mouse organs were harvested and plated on LB agar plates. (**D**) Phage counts were calculated from plaques on JJ2528. P-values were generated using a T-test statistical analysis. For phage counts (**D**), statistical analysis was done using one-way ANOVA and a Tukey’s test. Error bars represent standard deviation and “n” is equal to 15. One star (*) p < 0.05, two stars (**) p < 0.01, and three stars (***) p < 0.001.

A significant proportion of *E. coli* bacteremia originates in immunocompromised individuals. These patient populations constitute a sizable fraction of cancer, HIV, organ transplant, or otherwise vulnerable individuals whose immune function is low. In particular, cancer patients receiving chemotherapy are at risk of developing neutropenia, a condition characterized by a severe decline in circulating immune cells^34^. Prophylactic antibiotic treatments are necessary for these patients to prevent life-threatening infections, but such treatments may eliminate protective flora while inducing resistance^35^. To determine if phage are effective in this clinical context, we utilized a mouse model of chemotherapy-induced neutropenia whereby ExPEC-colonized mice undergo translocation from the gastrointestinal tract to develop a deadly bacteremia^36^. Mice were gavaged with JJ2528 and the animals were treated on alternate days with the cancer chemotherapy drug cytoxan (to induce neutropenia and bacterial translocation) and then given ϕHP3 (IP) (Fig. 5A). Fecal CFU demonstrated both mouse groups were colonized by JJ2528 at equivalent levels (Fig. 5B). Importantly, in mice treated with ϕHP3, there was a reduction in bacterial CFU in the assessed organs compared to the untreated mice. The majority of the untreated mice had bacteria in their liver and kidneys, while with ϕHP3 treatment, these organs showed a statistically significant (p = 0.03) reduction in CFU (~1 log difference). Only 2 out of 15 ϕHP3 treated mice had bacteria in the spleen (Fig. 5C,ii), which correlated with this organ also having the most (2.5 logs greater) PFU of phage (Fig. 5D). Interestingly, phage titers were detected in intestinal tissue although phage was administered parenterally (Fig. 5D).

## Discussion

Here, we report (i) the isolation of phages from avian and canine reservoirs, (ii) that all of the phages examined show morphological features consistent with being members of the *Myoviridae*, (iii) that they show broad lytic activity on a diverse array of multidrug-resistant ExPEC ST131 strains, (iv) that one of these isolates, ϕHP3, is efficacious against two human isolates in an injectional model of ExPEC bacteremia, (v) that a high bacterial burden *in vivo* and the *in vitro* lytic efficiency correlate with efficacy during bacteremia, and finally (vi) that these phage are effective against ExPEC in a model of gut-derived bacteremia in the immunocompromised host. This work demonstrates that it is quite feasible to isolate lytic phage with variable specificity for ExPEC and that these phages can have therapeutic efficacy in models of ExPEC bacteremia.

The structurally characterized phages described in this report are similar to the family of *Myoviridae*. The *Myoviridae* encompass five genera of bacteriophages, including the well-characterized Mu and T4-like viruses^37^,^38^. These phages include those with activity against two medically significant Gram-positive bacteria, including phage K which targets *Staphylococci*^39^ and phage EJ-1 which kills Streptococci^40^. Also included in this family are phage that target Gram-negative bacteria, such as *Pseudomonas* and Klebsiella^41^,^42^, as well as phage developed for the control of diarrheagenic bacteria such as *E. coli* O157:H7^43^. A recent paper described a bacteriophage from the *Podoviridae* family that could lyse O25b strains from ST131 and ST69 groups, and demonstrated some efficacy in infection models^44^. Work here describes three myophage isolates, as well as additional phage preparations from various environmental sources, that demonstrate broad lytic activity against a diverse sampling of ST131 ExPEC strains. Shared strain preference amongst viruses from two distinct environmental reservoirs may indicate that the ST131 ExPEC clonal group harbors a shared receptor that is recognized by these and related myophages, and that such phages are quite ubiquitous in the environment.

The crisis of lethal infections with multidrug-resistant gram negative bacteria is now being formally recognized by world leaders and health organizations^45^,^46^,^47^,^48^,^49^. It has its origin in (i) the overuse of antibiotics in the food and health industries^2^, (ii) the ability of *E. coli* to exchange genetic information, (iii) asymptomatic and chronic co-habitation in the gastrointestinal tract of human and domesticated hosts^1^,^26^ and (iv) medical treatments which make the human host susceptible to such infections, including cancer chemotherapy^34^. Chronic colonization and surface polysaccharide diversity makes universal vaccination against *E. coli* a challenge. The development of new antibiotics has not adequately kept pace with the rise of resistance, largely because antibiotic development is a lengthy process associated with sometimes prohibitive costs. Therapeutic phages have been suggested by many as a potential solution, in terms of both prophylaxis and treatment^50^.

In addition to being considered safe for human use^51^, the rapid evolution of phage allows for either expansion or restriction of the bacterial host range in days with simple laboratory procedures^52^,^53^,^54^ making it useful for clonal outbreak strains such as ST131 that emerge as dominant human pathogens^55^. Also, their lytic activity occurs by a different mechanism than killing by antibiotics and their selective pressure can lead to the emergence of bacterial populations that have re-acquired sensitivity to antibiotics^56^,^57^,^58^. Consistent with these ideas, we show here that suspected ExPEC reservoirs harbor phages that can efficiently kill clinically circulating and multi-drug resistant ST131 ExPEC. One of these viruses, ϕHP3, demonstrates the ability to significantly improve the health of mice infected with two virulent ExPEC strains and thus can lower the bacterial load below the threshold of bacteria needed to lead to a deadly infection. ϕHP3 can also circulate to major organ tissues, as evidenced by its high titers in liver, lung, spleen, and kidney and its appearance in the intestine when examined. The levels of ExPEC in these infections after a single dose of ϕHP3 treatment reduced the circulating levels of ExPEC by about 10,000 fold, activity which correlated with *in vitro* lytic results observed in culture and/or on agar plates. ϕHP3 demonstrated less efficient killing activity against strain JJ2547 in culture which correlated with a loss of ~1,000 fold killing potential in the treatment of mice infected with this strain. These findings indicate that the trends observed *in vitro* do reflect those *in vivo*; however, even a slight loss in activity can translate into a much greater loss in the context of a complex mammalian host. This complexity likely works against the infection optimum and amplifies subtle *in vitro* differences in mounting a productive and complete infection. Thus, it would seem reasonable to include as a part of the study of phage therapy a molecular understanding of the mammalian host factors that influence *in vivo* efficacy.

The propagation of phage in a mammalian host is bacterial density dependent^59^,^60^. The density of bacteria may in turn be dependent on their intrinsic virulence; thus, more virulent ExPEC strains that invade the blood and grow to high levels may be more susceptible to phage. Indeed, we observed a correlation between ExPEC virulence and the phage’s ability to reduce both health scores and bacterial burden. However, this creates a conundrum in that ExPEC that are less virulent but nevertheless a health concern may not be as responsive to phage. Infections with strains that do not produce robust bacteremia may require a combination treatment of antibiotics and phages or multiple treatments of phage over time. Combinatorial treatment with antibiotics can be beneficial by several different mechanisms. A resistance mutation to phage may be detrimental to the development of antibiotic resistance (or vice-versa), as strains are rarely resistant to both^61^. Also, these mutations could lead to fitness costs rendering them non-competitive *in vivo*^59^. Thus, it would seem that a comprehensive evaluation of phage therapy should also include the combinatorial use of phage and antibiotics, perhaps as a type of “adjuvant” to antibiotics.

Phage used to prevent or treat ExPEC infections is not without scientific and clinical barriers. The most serious obstacle is bacterial resistance to phage infection^62^. Prolonged (>24 hours) incubation of ExPEC strain JJ2528 with ϕHP3 did result in a low-level of bacterial cells that become resistant to lytic killing (data not shown). Another barrier to phage efficacy is the human immune system. The production of anti-phage antibodies in response to repeated dosage of phage over long periods is well-documented^63^,^64^,^65^. The antibody response to phage phiX174 is so robust that it is used to test immune responsiveness in humans^66^. Normal antibody responses to this antigen are consistently defective in patients with HIV^67^, genetic immunodeficiencies^68^ and receiving immunosuppressing treatments^69^. In a clinical study of patients treated with phage therapy for bacterial infections, in which 50% of the patients were immunocompromised, 60% of patients given local administration of phage demonstrated low antibody neutralization in serum^70^. This is interesting because in our study we chose to test phage treatment of a bacterial infection within the context of immunosuppression, which is when patients are most at risk of these opportunistic ExPEC infections. The pronounced reduction of bacterial counts in all organs of treated mice in this model of bacterial translocation suggest phage therapy should be considered as one possible solution for these types of infections in high risk, immunocompromised patients. Such a therapy may prove useful in this patient population as there may be a reduction in circulating antibodies following phage treatment which would allow for repeated dosing and hence greater efficacy. Regardless, the fact that such efficient killers of ExPEC can be isolated and tested in such a short period of time indicates that phage should be considered an “adaptive drug”, evolved in real-time to meet the specific needs of the problem at hand, thereby keeping pace with the rates at which resistance, or neutralization, arises.

## Materials and Methods

### Experimental Animals

The mouse strain BALB/c (Jackson laboratories, Bar Harbor, ME) was used for Fig. 3 and Swiss Webster (Charles River Wilmington, MA) for Figs 4 and 5. All mice were 6 weeks of age and female. They received sterile food and water ad libitum and were housed individually in filtered cages post-infection or gavage. All methods performed on mice were approved in accordance with relevant guidelines and regulations from “The Guide and Care and Use of Laboratory Animals” (National Institute of Health) and approved by Baylor College of Medicine’s Institutional Animal Care and Use Committee (AN-5177).

### Bacterial Strains and Growth Conditions

ExPEC strain CP9^36^ and MG1655 K12 were used as phage isolation strains. *E. coli* MG1655 (λ 16831) was used for the lysogeny assays. Figure 1 lists other *E. coli* strains (kindly provided by Dr. James Johnson, University of Minnesota) that were permissive for phage infection. All strains were grown in Lysogeny Broth (LB) at 37 °C from individual colonies selected on plates after resuscitation from a frozen stock (−80 °C, 10% glycerol).

### Phage Isolation and Plaque Assay

Geese, duck, and dog feces were collected from two parks (2 miles apart) in the Houston, Texas area. Chicken feces were collected from a private farm. Fecal samples were homogenized in phage buffer (1.0 M NaCl, 0.01 M Tris, pH 8.0, 0.001 M EDTA) by rocking gently using a benchtop rocker, separated by a Sorvall Lync 4000 Lynx centrifuge (Thermo Scientific, Waltham, Mass) at 13 K rpm (~29000 g, 50 min) and the supernatant filter sterilized (0.22 μm). The filtrate was added to a soft agar overlay assay containing the isolation bacterial strain. Plaques of different morphology were selected, grown to high titer in the isolation strain and the lysates used to generate the efficiency of plating (EOP’s) presented in Fig. 1.

### Phage Purification

Small batch cultures of CP9 for ϕCF2 and JJ2547 for ϕHP3 were grown to an O.D. of 0.1 at 600 nm, infected with phage lysate (MOI 0.1) until complete lysis was achieved and used to infect a large batch culture prepared in 4 Liters of LB. The lysate was centrifuged at 10 K RPM (~17000 g, 4 °C, 30 min) and precipitated with 30 g NaCl/1000 mL and 300 g of Polyethylene Glycol 8000. After overnight precipitation at 4 °C, the phage was pelleted at 10 K RPM (17000 g, 4 °C, 30 min) and then resuspended in 40 mL of phage buffer. One volume of CHCl_3_ was added to the resuspended phage, emulsified, and phases separated by centrifugation, 10 K RPM (17000 g, 10 min). Cesium Chloride (CsCl) was added to the aqueous phase, treated with 5 μg/ml DNAse/RNAse mix for 30 minutes at 37 °C, adjusted to a density of 1.5 and ultracentrifuged using SW41 rotor (20 K RPM, 10 °C, 18 hrs). The phage band was collected and dialyzed in phage buffer (2000X Vol, twice).

### Lysogeny testing

A lysate consisting of ϕHP3 was added (MOI 10) to stationary phase *E. coli* JJ2528 in an LB overlay assay. The next day, bacterial colonies insensitive to ϕHP3 were isolated from these plates and were re-plated to remove residual phage. The induction of lysogens was tested using the method described for Zeng *et al*.^30^. Insensitive colonies from strains *E. coli* JJ2528 and *E. coli* MG1655 (λ 16831) were grown to log phase in LB. Cultures were incubated with the DNA damaging agent mitomycin C (10 μg/ml, Fisher BioReagents, Waltham, Mass.) for two hours. Chloroform (2%) was added to the flasks to lyse cells. Lysed cultures were pelleted via centrifugation (7,000 × G, 10 minutes) and an aliquot removed and serially plated on a lawn of the originator strain. Other ϕHP3 insensitive colonies, *E. coli* JJ2528 and *E. coli* MG1655 (λ 16831) were lysed after log phase growth and pelleted to plate for the spontaneous induction of phage.

### Measurement of burst size and latent period

These procedures were based on the experiments of Ellis and Debruick^71^ with some modifications^72^. An overnight culture of *E. coli* JJ2528 was grown to mid-log phase and purified ϕHP3 was added at an MOI of 1. Phage was allowed to adsorb for 20 minutes with shaking (255 rpm, 37 °C). Following adsorption, the culture was pelleted via centrifugation (10,000 × G, 5 minutes). The pellet was washed and prewarmed LB media added to resuspend the pellet. The resuspended culture was grown as before (37 °C, 255 rpm). Samples (100 μL) were taken every 10 minutes after resuspension for 2 hours total. Samples were lysed (Chloroform 2–5%) and pelleted (8,000 × G, 5 minutes), supernatant aliquots were serially diluted and plated on a lawn of *E. coli* JJ2528. ϕHP3 burst size was calculated as the final titer of the plateau period to the initial titer of infected bacteria^73^. The latent period was determined to be the interval when there was no or little increase in the titer^73^.

### Adsorption Assays

Adsorption was measured according to established methods^72^. Briefly, ϕHP3 (MOI 1) was added to a mid-log phase culture of *E. coli* JJ2528. Immediately after addition of ϕHP3, 100 μl of culture was removed (time point “0”). This sample was added to 900 μL of chilled LB media (choroform added) then vortexed and kept on ice. This was repeated with samples taken every minute for a total of 10 minutes. All samples were pelleted via centrifugation (8,000 × G, 5 minutes) and supernatant aliquots were serially diluted and plated on a lawn of JJ2528. An adsorption curve was generated using this data with the adsorption rate constant calculated as the natural log of the slope of the graph versus the bacterial titer^72^.

### Bacteriophage sequencing and genome assembly

Phages HP3, EC1, and CF2 were grown to high titer and purified as described above. Phage DNA extraction, sequencing and assembly was done by the Center for Metagenomics and Microbiome Research (CMMR) at Baylor College of Medicine. Phage DNA was extracted using the Applied Biosystems MagMAX *Viral* DNA/RNA Isolation kit (Applied Biosystems, Foster City, CA) and eluted in 50 μl of Elution Buffer. Extracted DNA samples were constructed into Illumina paired-end libraries. DNA from phages HP3, EC1, and CF2 were sheared into fragments of approximately 500–600 base pairs using a Covaris E210 system (Covaris, Inc. Woburn, MA). Products were then amplified through Ligation Mediated-PCR (LM-PCR), which was performed using the KAPA HiFi DNA Polymerase (Kapa Biosystems, Inc., Cat. no. KM2602). Purification was performed with Agencourt AMPure XP beads after each enzymatic reaction. Following the final XP bead purification, quantification and size distribution of the LM-PCR product was determined using the Agilent Bioanalyzer 7500 chip. The libraries had an average final size of 660 bp (including adapter and barcode) and were pooled in equimolar amounts to achieve a final concentration of 10 nM. The library templates were prepared for sequencing on an Illumina MiSeq. Briefly, this library was denatured with sodium hydroxide and diluted to 6 pM in hybridization buffer in order to achieve a load density of 670 K clusters/mm2. The library pool was loaded on a MiSeq flow cell which was spiked with 1% phiX control library for run quality control. The sample then underwent bridge amplification to form clonal clusters, followed by hybridization with the sequencing primer. Sequencing runs were performed in paired-end mode using the 600V3 kit. Sequencing-by-synthesis reactions were extended for 301 cycles from each end, with an additional 10 cycles for the index read. After sequencing, the.bcl files were processed through Illumina’s analysis software (CASAVA), which demultiplexes pooled samples and generates sequence reads and base-call confidence values (qualities). The average raw yield per sample was 3,064 Mbp. Raw data files were trimmed using Babraham Bioinformatics’ TrimGalore script^74^, and then had human and PhiX removed using a combination of Bowtie^75^ and PRINSEQ^76^ against the Genome Reference Consortium Human Build 38 (HG38) assembly. Phage genomes were assembled using SPAdes Genome Assembler^77^ and verified on a Platanus Assembler^78^ which generated 1 complete contig per genome. Genome annotation was done using DNA Master. This program concurrently runs a collection of programs, including GLIMMER^79^ V. 3.02 for the prediction of open reading frames or ORFs and GeneMarkS^80^, to verify the calling of ORFs. ARAGORN^81^ V. 1.1 was used to predict tRNAs and BLASTP searches were performed on predicted ORFs for a functional analysis. Bacterial virulence genes were searched using VirulenceFinder^29^ and genes encoding antibiotic resistance using ResFinder^31^.

### Cryo-electron Microscopy

Quantifoil grids of mesh size 400 (Quantifoil Micro Tools GmbH, Großlöbichau, Germany) were rendered hydrophilic by 20–25 s glow discharge, or in the case of phage CF2, by 5 s plasma discharge in a Solarus Model 950 (Gatan, Pleasanton, CA). Next, 3 μL of phage were applied to the grid, blotted for 1–2.5 s, and vitrified in a Vitrobot Mk IV (FEI, Hillsboro, OR). Grids were imaged in JEOL 200 kV cryoelectron microscopes and images were recorded with a DE12 direct detection device (Direct Electron LP, San Diego, CA) or with a US4000 CCD (Gatan). To render figures, images were low-pass filtered to a resolution of 20 Å using EMAN2^82^. Feature lengths were measured manually in EMAN2 based on calibrated detector pixel sizes.

### Murine Infections

All methods described below were approved in accordance with relevant guidelines and regulations by the Institutional Animal Care and Use Committee.

#### Injectional bacteremia model

Overnight bacterial cultures were grown to log phase at 37 °C (250 rpm). Cultures were washed twice in 1XPBS. Colony forming units (CFU) were determined by plating on bacteriologic agar and a dose of 10^8^ CFU injected intraperitonealy (IP)^83^. After 1 hour, infected mice were injected with purified ϕHP3, IP, with 10^9^ plaque forming units - PFU in 100 μl of PBS.

#### Translocation bacteremia model

This model system is described in detail in reference^36^. Briefly, mice were gavaged with 10^9^ CFU of ExPEC strain JJ2528 and animals administered cyclophosphamide (Cytoxan, Baxter Healthcare Corporation, Deerfield, IL, USA) IP for a total dose 450 mg/kg body weight on days 1, 3 and 5 as indicated in Fig. 3. Purified ϕHP3 was administered IP on the days 2, 4 and 6 as indicated in Fig. 3 and the mice necropsied on day 7 post infection.

#### Measurement of health scores

The scale for measuring murine health is based on four scored parameters, including activity (lethargy), grooming behavior (rough coat), body condition (hunched posture), and respiratory distress (hypernea or labored breathing), as outlined by the NIH Animal Research Advisory Committee Guidelines for the evaluation of rodent health^84^. Each observation of one of these criteria during a 20 minute period receives a score of 0, 0.5 or 1 depending on the severity. Mice with respiratory distress receive a score of 0 for normal breathing or a score of 1 for labored breathing (hyperpnea). All other parameters receive either a 0 (no disease), 0.5 (moderate) or 1 (severe). The scoring is performed by a blinded investigator that is trained to recognize these symptoms. When animals reach a score of 4, they are considered moribund and must be sacrificed according to our protocol approved by our institutional review board.

### Bacterial and phage quantification of organ and fecal homogenates

Immediately following euthanization mice were necropsied under sterile conditions. Lungs, kidneys, liver and spleen were weighed and homogenized in 1XPBS with sterile blades. Organ homogenates were serially diluted and plated on LB plates and incubated at 37 °C overnight. Phage counts were quantified using a soft agar overlay assay. Fecal counts were determined on LB and Ampicillin, 100 μg/ml, plates to prevent growth of other commensal flora.

### Statistics

Statistical significance values were determined using “R” version 3.2.4. For Fig. 3 panel B, and Fig. 4 panels A-i,B-i and C-i, a Wilcoxon rank sum test was used to assess significance. For Fig. 3 panel C, Fig. 4 panels A,ii–v,B,ii–v and C,ii–v and Fig. 5 panels B and C a two-tailed T-test on normalized data (log transformed) was used to test significance. For Figs 3D, 4D and 5D a One-Way ANOVA test was performed on normalized data and Tukey’s test for multiple comparison was used to determine significance. Unless otherwise noted, alpha values were set to 0.05 and statistical significance was determined if calculated P values were below 0.05. Figure “n” values are reported in the legend under each figure.

## Additional Information

**How to cite this article:** Green, S. I. *et al*. Bacteriophages from ExPEC Reservoirs Kill Pandemic Multidrug-Resistant Strains of Clonal Group ST131 in Animal Models of Bacteremia. *Sci. Rep.* **7**, 46151; doi: 10.1038/srep46151 (2017).

**Publisher's note:** Springer Nature remains neutral with regard to jurisdictional claims in published maps and institutional affiliations.

## Figures and Tables

**Table 1:** Genomic characterization of phage HP3, CF2, and EC1.

Bacteriophages (Φ)	Size (bp)	G + C (%)	ORFs	tRNAs	Accession #
ϕHP3	168,188	35.4	274	11	KY608967
ϕCF2	53,242	45.9	74	0	KY608966
ϕEC1	170,254	37.6	275	2	KY608965
ϕpSs-1 (Like ΦHP3)^A^	164,999	35.5	266	10	KM501444.1
ϕBP63 (Like ΦCF2)^B^	52,437	46.0	76	0	NC_031250.1
ϕSHSML-52-1 (like ΦEC1)^C^	169,621	37.6	269	2	KX130865.1

^A^Shigella phage ϕpSs-1 has 96% nucleotide homology to ϕHP3.

^B^Salmonella phage BP63 has 96% nucleotide homology to ϕCF2.

^C^Shigella phage SHSML-52-1 has 98% nucleotide homology to ϕEC1.

Characterization includes genome size (base pairs or bps), percent G + C content, open reading frames (ORFs) and tRNAs.

**Table 2:** Basic characterization of phage HP3.

Adsorption		Growth Curve		Plaques	
Constant (K)	%	Burst Size	Latent Period	Size	Morphology
5.63 × 10^−9^ ML/Min^a^	99.8^B^	60 ^C^	22.5 min^D^	0.5 mm	Clear

Characterization of phage HP3 includes calculated adsorption constant (K), percent phage adsorbed, burst size, latent period, plaque size and morphology. All experiments were performed using *E. coli* JJ2528 as host.
